# Supplementary material for: Porous supraparticle assembly through self-lubricating evaporating colloidal ouzo drops
Source: Nat Commun. 2019 Jan 29;10:478. doi: 10.1038/s41467-019-08385-w (PMC6351649; doi:10.1038/s41467-019-08385-w)
Supplement: Supplementary file 2 — Description of Additional Supplementary Files [file 41467_2019_8385_MOESM2_ESM.pdf]

Description of Additional Supplementary Files

Supplementary Movie 1: Porous supraparticle assembly through evaporating colloidal ouzo drops.
The drop solution consists of 39.75 vol% water, 59.00 vol% ethanol, 1.20 vol% trans-anethole and 0.05 vol% TiO_2 nanoparticles.

Supplementary Movie 2: The confocal movie shows the self-lubrication process during the supraparticle self-assembly in the case of low oil-to-nanoparticle ratio. Color indications under a confocal microscope: yellow, oil; blue, water/ethanol; black, clusters of nanoparticles; red, substrate.

Supplementary Movie 3: The confocal movie shows the self-lubrication process during the supraparticle self-assembly in the case of high oil-to-nanoparticle ratio. The color indications are same as Movie S2.

Supplementary Movie 4: Slide-by-slide cuts by FIB to reveal the inner structure of the supraparticle.

Supplementary Movie 5: The nucleated oil microdroplets in a colloidal ouzo drop coalesce during the evaporation. The nanoparticle concentration is less than 0.0005 vol%. It reveals that the oil microdroplets act as cells devoid of (clusters of) nanoparticles.

Supplementary Movie 6: The movie shows the automatic production of drops of similar size on the OTMS surface with our setup.

Supplementary Movie 7: The detachment of the generated supraparticles from the OTMS surface by rinsing with ethanol drops.

Supplementary Movie 8: The detachment of the generated supraparticles from the OTMS surface by dipping inside the ethanol solution.

Supplementary Movie 9: Colloidal ouzo drop created by spray, to fabricate large quantities of supraparticles (without controlled sizes) .
